# Aryl Hydrocarbon Receptor Modulates Carcinogenesis and Maintenance of Skin Cancers

**DOI:** 10.3389/fmed.2019.00194

**Published:** 2019-09-04

**Authors:** Takanori Hidaka, Taku Fujimura, Setsuya Aiba

**Affiliations:** Department of Dermatology, Tohoku University Graduate School of Medicine, Sendai, Japan

**Keywords:** aryl hydrocarbon (Ah) receptor, squamous cell carcinoma, melanoma, ultraviolet, air pollutant, BRAF inhibitor, PD-1

## Abstract

The aryl hydrocarbon receptor (AHR) is a ligand-activated transcription factor that responds to a wide range of chemicals, including chemical carcinogens such as dioxins and carcinogenic polyaromatic hydrocarbons, and induces a battery of genes associated with detoxification, proliferation, and immune regulation. Recent reports suggest that AHR plays an important role in carcinogenesis and maintenance of various types of skin cancers. Indeed, AHR is a susceptibility gene for squamous cell carcinoma and a prognostic factor for melanoma and Merkel cell carcinoma. In addition, the carcinogenic effects of ultraviolet (UV) and chemical carcinogens, both of which are major environmental carcinogenetic factors of skin, are at least partly mediated by AHR, which regulates UV-induced inflammation and apoptosis, the DNA repair system, and metabolic activation of chemical carcinogens. Furthermore, AHR modulates the efficacy of key therapeutic agents in melanoma. AHR activation induces the expression of resistance genes against the inhibitors of V600E mutated B-Raf proto-oncogene, serine/threonine kinase (BRAF) in melanoma and upregulation of programmed cell death protein 1 (PD-1) in tumor-infiltrating T cells surrounding melanoma. Taken together, these findings underscore the importance of AHR in the biology of skin cancers. Development of therapeutic agents that modulate AHR activity is a promising strategy to advance chemoprevention and chemotherapy for skin cancers.

## Introduction

Recently, the incident rate of skin cancer has been greatly increasing. The number of patients treated for skin cancers has increased by 44% during the past 5 years (1), and skin cancer has become the most common cancer type in Caucasians (2). Although both genetic and environmental factors contribute to the carcinogenesis of skin cancer, this rapid increase suggests the relative importance of environmental factors. The skin is the outermost interface between the body and the environment and is ineluctably exposed to environmental insults such as ultraviolet radiation (UVR) or air pollutants (3). As UVR and air pollutants can induce carcinogenesis in the skin (4), the skin contains a system that recognizes and detoxifies these carcinogenic insults, the dysregulation of which leads to the initiation of skin cancer. In addition to the increase in carcinogenesis of skin cancer, recent therapeutic aspects of skin cancer have greatly changed. In particular, the emergence of molecular targeted therapies including inhibitors for V600E mutated B-Raf proto-oncogene, serine/threonine kinase (BRAF) and checkpoint inhibitors, which attenuate suppression of the anti-tumor immune response, have drastically improved the outcome of advanced melanoma. These drugs retrogradely elucidated the critical contribution of specific proliferative signals and tumor immunity in the maintenance of melanoma. These recent changes in skin cancers imply the importance of identifying a key molecule that modulates carcinogenesis and maintains skin cancer to improve prevention of and therapy for skin cancers.

**Figure 1:**
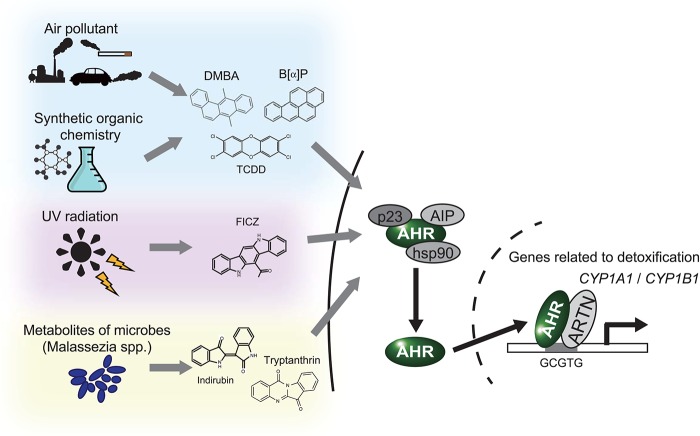
AHR works as an environmental sensor. AHR binds to polycyclic aromatic hydrocarbons and their derivatives derived from environment. Once these ligands binded, AHR isolates from the complex in cytoplasm, translocates into nucleus and activates translation of the target genes, including CYP1A1 and CYP1B1. DMBA, 7,12-Dimethyl benz[*a*]anthracen; B[*a*]P, Benzo[*a*]pyrene; TCDD, 2,3,7,8-Tetrachloro dibenzo-*p*-dioxin; FICZ, 6-Formylindolo [3,2-*b*]carbazole.

**Figure 2:**
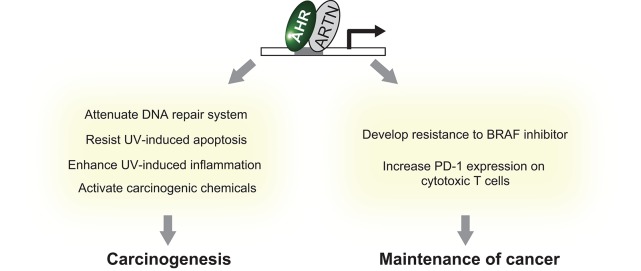
Summary of the effect of AHR activation on skin cancer.

The aryl hydrocarbon receptor (AHR) is an evolutionarily conserved, ligand-activated transcription factor, which is a member of the basic helix-loop-helix/PER-ARNT-SIM family (5). Due to its broad capacity to recognize a wide range of chemicals in the environment, AHR is often described as an environmental sensor. Once activated by ligand binding, AHR translocates into the nucleus and dimerizes with ARNT (Ah receptor nuclear translocator). Then the AHR/ARNT heterodimer enhances the expression of its target genes that encode drug-metabolizing cytochrome P450s, including *CYP1A1, CYP1A2*, and *CYP1B1* (6) (Figure 1). These target molecules of AHR facilitate the metabolic degradation of its ligands. In addition to this role in detoxification, recent works have also revealed novel roles for AHR in tumor biology. In various tumors, differential expression of AHR is indeed observed compared to normal tissue. This different expression status of AHR plays a critical role in pro- or anti-tumor activity according to the cell state (7). Regarding skin cancer, a genome-wide association study of cutaneous squamous cell carcinoma (SCC) also identified *AHR* as a novel susceptibility locus (8). Furthermore, among various solid tumors, the expression level of *CYP1A1, CYP1A2*, and *CYP1B1* is associated with prognosis of melanoma (9). These findings imply that AHR also plays important roles in the biology of skin cancers. In support of this hypothesis, AHR has recently been found to be associated with UVR and air pollutant-induced carcinogenesis of skin cancer (10, 11). Furthermore, AHR may play a role in modulating the efficacy of BRAF inhibitors and checkpoint inhibitors (12, 13) (Figure 2). In the following sections, we introduce the function of AHR in the context of carcinogenesis and maintenance of skin cancer and mainly focus on environmental carcinogens and molecular targeted therapy.

## Environmental Factor-Induced Skin Carcinogenesis via AHR Activation

### Ultraviolet Radiation

As much as 90% of non-melanoma skin cancers are associated with exposure to UVR (14). UVR causes mutagenesis of DNA and inflammation, which may eventually lead to the formation of skin cancers. UVA radiation (400-320 nm wavelength) excites endogenous chromophores and generates reactive oxygen species, leading to modifications of oxidative bases and generation of 7,8-dihydro-8-oxoguanine at guanine bases (15). In contrast, UVB radiation (320-290 nm wavelength) activates a photochemical reaction and forms photoproducts, including cyclobutane pyrimidine dimers (CPDs) and pyrimidine 6-4 pyrimidones, at adjacent pyrimidine nucleotides (15). To keep genomic integrity, these DNA photoproducts have to be removed by DNA repair system or apoptosis, depending on the extent of DNA damage (16). However, once the incorrect repair of these DNA modifications occurs, it may inhibit polymerases, lead to the arrest of replication or cause misreadings during transcription or replication, which results in the formation of mutations, initiation of carcinogenesis, and skin cancer (15). The importance of DNA repair enzymes is clearly evident as seen by the drastically increased risk of developing UV signature mutations and subsequent skin cancers in Xeroderma Pigmentosum patients who lack one of the DNA repair enzymes (17). In addition to mutagenesis, UVR causes the body a UV stress response, the inflammatory response at the exposed site. Increasing evidence suggests that sustained inflammation induced by UVR plays an important role in cancer initiation and progression (18).

AHR acts as a light sensor in keratinocytes following activation by UVR. UVR (in particular UVB) generates formylindolo (3,2-b)carbazole (FICZ), a tryptophan derivative, in epidermal keratinocytes (19). FICZ functions as a high-affinity ligand for AHR and induces UVR-mediated AHR activation, which is associated with UV-induced skin carcinogenesis. Pollet et al. reported that the chronic irradiation of UVB causes only a half numbers of cutaneous SCCs on AHR^−/−^ mice compared to AHR^+/+^ littermates, which implies a critical contribution of AHR in carcinogenesis of SCC. As a molecular mechanism, they revealed AHR activation attenuates the clearance of UVB-induced CPDs by repressing global genomic repair in a p27-dependent manner (10). In addition to the role of AHR in the attenuation of DNA repair systems, AHR works as a negative regulator of apoptosis in UVB damaged keratinocytes. Frauenstein et al. reported that chemical inhibition or knockdown of AHR sensitize keratinocytes to UVB-induced apoptosis by decreasing the expression of E2F1 and its target gene checkpoint kinase 1 (CHK1) (20). AHR also promotes the UV stress response (19). Although the concise molecular mechanism of the UV stress response remains largely unknown, the involvement of different tyrosine kinases including the epidermal growth factor receptor (EGFR) and pro-inflammatory molecules has been suggested (21). For instance, FICZ induces the internalization and subsequent activation of EGFR in an AHR-dependent manner, which is also induced by UVB radiation (19). Moreover, AHR activation in keratinocyte induces the expression of various pro-inflammatory molecules. Irradiation of UVR followed by topical application with FICZ on *Ahr*^+/+^ mice induces cutaneous expression of a neutrophil directing chemokine (C-X-C motif) ligand 5 (Cxcl5) compared with UV alone, which cannot be observed in *Ahr*^−/−^ mice (22). In addition, the exposure of FICZ to keratinocyte cell-line induces the activation of AHR and ROS production, which leads to the production of pro-inflammatory cytokine, IL-6 (23). Furthermore, AHR activation induced by UVB irradiation can activate the expression of cyclooxygenase-2, which is pro-inflammatory and associated with the development of skin cancer (19, 24).

These observations imply that AHR activation promotes UVR-induced skin carcinogenesis via attenuation of the DNA repair system and apoptosis and via enhancement of the UV response.

### Carcinogenic Chemicals in Air Pollutants

Carcinogenic chemicals are another well-known type of environmental carcinogen that leads to skin cancer. Airborne particulate matter (PM) and ambient air pollution, which contain various carcinogenic chemicals, are considered group 1 human carcinogens by the International Agency for Research on Cancer (25, 26). As the skin is located at the outermost layer of the body, it is continuously exposed to air pollutants, which may increase the risk of skin cancer. Carcinogenic chemicals, including polycyclic aromatic hydrocarbons (PAHs) and dioxins, contained in PM are responsible for PM-induced carcinogenesis (27). Due to their lipophilicity, these chemicals easily penetrate through the skin (28, 29) and are retained in the skin for a long time (30). PAHs and dioxins exert their biological effects via binding to AHR. AHR activation by these chemicals has gained a lot of attention as a mechanism that contributes to skin carcinogenesis. In fact, PAHs and dioxins cause SCC in *in vivo* animal models. For instance, chronic subcutaneous injection of TCDD to hamster results in formation cutaneous SCC (31). In addition, application of 7,12-Dimethylbenz[*a*]anthracene (DMBA), a member of the PAH family that is typically found in cigarette smoke, to murine skin causes lesions that are histologically similar to benign papilloma to SCC (32, 33). Whole-exome sequencing analysis has been conducted in this murine model of DMBA-induced SCC to investigate its mutational landscape (34). As a result, the majority of DMBA-induced SCC possesses mutations in oncogenes including *Hras, Kras*, and *Rras2*. These mutations in human SCCs are similar to those in head and neck, esophageal, lung, and cervical SCC (34–36). In addition to SCC, the development of melanoma is also accelerated by the application of DMBA in some genetically engineered mouse models of melanoma (37).

In these models of PAH-induced skin carcinogenesis, AHR plays a considerable role. Chronic topical application of organic extracts of airborne particulate matter causes SCCs in a half of AHR ^+/+^ mice but none of AHR^−/−^ mice (11). Benzo[*a*]pyrene, another PAH contained in PM from cigarettes or air pollutants, can also induce SCC following subcutaneous or topical application to wild-type mice. This carcinogenic property of benzo[*a*]pyrene is attenuated when applied to *Ahr*-deficient mice (38). In the case of DMBA-induced carcinogenesis, the mice possessing the 375A allele of *Ahr*, encoding the high-affinity ligand-binding receptor, develop skin cancers, but the mice possessing the 375V allele, encoding the low-affinity one do not (39); in contrast, there is another report demonstrating no significant differences in carcinogenesis between *Ahr*^+/+^ mice and *Ahr*^−/−^mice by topical application of DMBA (40). Taken together, these results suggest that AHR activation promotes tumor induction of PAH-induced skin carcinogenesis.

Several studies investigated the mechanism of PAHs-induced carcinogenesis and revealed that AHR-dependent induction of CYP1A1/CYP1B1 expression likely plays a key role (38). In general, CYP1A1 and CYP1B1 enzymes facilitate removal of AHR ligands by degrading them to metabolites with decreased activity and increasing their water solubility (41). In contrast, in the case of carcinogenic PAHs, the same metabolic reaction results in the metabolic activation of PAHs. For instance, CYP1B1-mediated metabolism of DMBA results in the synthesis of DMBA-trans-3,4-diol, which is highly electrophilic and causes damages to DNA (42). Moreover, CYP1A1, CYP1B1 and epoxide hydrolase-mediated metabolism of benzo[*a*]pyrene results in the synthesis of highly electrophilic benzo[*a*]pyrene-7,8-diol-9,10-epoxide (43).

Regarding the mechanism of dioxins-induced carcinogenesis and its dependency of AHR, they remain largely unknown, as dioxins are not generally metabolized to be directly genotoxic and there is lack of related articles.

## Maintenance of Skin Cancer via AHR Activation

In addition to the carcinogenic role of AHR activation, AHR also greatly contributes to the maintenance of various skin cancers. In non-cutaneous tumors, AHR is an established factor that induces suppression of the anti-tumor immune response, resulting in the escape of tumor cells from immune-mediated cell death (44). Furthermore, AHR affects multiple aspects of cancer biology, including cell survival and proliferation (45). Recent findings show that AHR modulates anti-tumor immunity and proliferative signals in skin cancers. In the following sections, we introduce recent findings regarding how AHR contributes to the maintenance of skin cancers, mainly focusing on melanoma.

### Melanoma

Melanoma is believed to be derived from malignant transformation of melanocytes, which are pigment-producing cells that generally reside in skin (46). Studies investigating the melanocytes of *Ahr*-deficient mice indicated that AHR is essential for proliferation of melanocytes (47). In addition, some reports using melanoma cell lines indicate that AHR activation attenuates tumorigenicity (48, 49); in contrast, others reported that AHR activation promotes tumorigenicity of melanoma (50, 51). These observations suggest the contribution of the AHR system to the biology of melanoma, the details of which have been revealed in recent reports.

In the clinical setting, therapy for melanoma is based on the staging system, which scores clinical and pathological risk factors, including tumor thickness, mitotic rate, and presence of ulceration and metastases (52). In the past, once a melanoma was scored as high grade, patients were considered to have an extremely high mortality rate due to resistance to chemotherapy (53). However, recent development of molecular targeted therapies against the oncogene or checkpoint inhibitors has drastically improved the prognosis of patients with advanced melanoma (54). This improvement indicates the critical importance of BRAF and checkpoint molecules in the maintenance of melanoma. Surprisingly, recent findings have revealed a significant role for AHR in modulating the effect of these critical molecules.

The BRAF V600E mutation is the most prevalent mutation and is present in approximately half of patients with advanced melanoma (55). Specific inhibitors of mutated BRAF have achieved high response rates and improved overall survival (56). Meanwhile, the efficacy of BRAF inhibitors is transient due to acquired resistance, which usually appears within a year after the time of response and results in relapse of melanoma (57, 58). One mechanism of the induction of resistance to BRAF inhibitors is upregulation of genes related to resistance to BRAF inhibitors, including AXL receptor tyrosine kinase (*AXL*), *EGFR*, and neuropilin 1 (*NRP1*) (59, 60). Recently, Corre et al. demonstrated that in a subset of melanoma cells, AHR is constitutively activated, which drives expression of these genes that are related to resistance to BRAF inhibitors (12). In addition, they also reported that co-administration of AHR antagonists with BRAF inhibitors maintains at least partial sensitivity to BRAF inhibitors in melanoma cells.

Melanoma is a solid tumor with a high mutational burden, which induces the generation of neo-antigens and the infiltration of cytotoxic T cells (CTLs) that recognize neo-antigens (61, 62). The level of mutational burden is correlated with that of transcripts related to cytolytic activity of local immune infiltrates (63). To evade the anti-tumor immune response, melanoma cells express molecules associated with checkpoint pathways. Approximately 40% of melanoma biospecimens express programmed death-ligand 1 (PD-L1), one of the molecules associated with the checkpoint pathway (64). When PD-L1 expressed on melanoma cells binds to the PD-1 receptor expressed on CTLs, CTLs become dysfunctional, and melanoma cells escape immune-mediated cell lysis (65, 66). As mentioned, PD-1 blockade by checkpoint inhibitors significantly improves overall survival and progression-free survival compared with classical chemotherapy in patients with advanced melanoma (67). These findings imply the importance of elucidating how melanoma cells upregulate the expression of PD-1 on CTLs. Liu et al. found that tumor-repopulating cells, a subpopulation of cancer cells having stem cell-like property that are tumorigenic and can grow in soft 3D matrices, produce kynurenine, a known AHR ligand of tryptophan metabolism, by type I IFN-induced expression of indolamine 2,3-dioxygenase. Then kynurenine activates AHR in tumor-repopulating cells, which enters them into dormancy, the condition resistant to immune-therapies (68, 69). In addition, released kynurenine is taken up by surrounding CTLs and upregulates PD-1 expression on CTLs in an AHR-dependent manner (13). This finding tells us that the AHR system may be a significant modulator of PD-1-mediated suppression of the anti-melanoma immune response.

### Other Cutaneous Carcinomas

Several reports have suggested possible links between the AHR system and tumor biology in Merkel cell carcinoma (MCC) and extramammary Paget's disease (EMPD).

MCC is a rare and aggressive neuroendocrine skin cancer, and ~80% of patients are infected with merkel cell polyomavirus. Univariate analysis of clinical specimens revealed that a longer overall survival is achieved in the group with lower expression of tryptophan 2,3-dioxygenase 2 (TDO2) and AHR in cells surrounding the tumor (70). As TDO2 is an enzyme in the tryptophan-kynurenine metabolic pathway, the TDO2-AHR axis may play a significant role in the pathophysiology of MCC.

Another study of EMPD, an adenocarcinoma of apocrine origin, reported that the epidermis adjacent to EMPD lesions expresses CYP1A1 and CCL20, an interleukin-17-related chemokine. Malassezia yeast, which are often pathogenic in apocrine lesions, produce a metabolite that activates AHR and induces the Th17 immune response. Thus, a possible link may be present between Malassezia metabolite-induced AHR activation and the Th17-skewed tumor immune response in EMPD (71).

## Concluding Remarks

As summarized above, AHR was recently found to be a key modulator of UVR- and carcinogenic chemical-induced skin carcinogenesis. In addition, this molecule is associated with the efficacy of BRAF inhibitors and checkpoint inhibitors, which are core therapeutic drugs in melanoma. Taken together, these data underscore the importance of the AHR system in carcinogenesis and maintenance of skin cancers, especially SCC and melanoma. This means that the AHR system is a putative target, particularly for chemoprevention and cancer chemotherapy of skin cancer. The emergence of research investigating the effect of AHR antagonists for various skin cancers is promising and eagerly awaited.

## Author Contributions

TH wrote the manuscript. TF and SA supervised and reviewed this work.

### Conflict of Interest Statement

The authors declare that the research was conducted in the absence of any commercial or financial relationships that could be construed as a potential conflict of interest.

## References

[1] Guy GP Machlin SR Ekwueme DU Yabroff KR. Prevalence and costs of skin cancer treatment in the U.S., 2002–2006 and 2007–2011. Am J Prev Med. (2015) 48:183–87. 10.1016/j.amepre.2014.08.036 25442229 PMC4603424

[2] Leiter U Eigentler T Garbe C. Epidemiology of skin cancer. Adv Exp Med Biol. (2014) 810:120–40. 10.1007/978-1-4939-0437-2_7 25207363

[3] Elias PM Choi EH. Interactions among stratum corneum defensive functions. Exp Dermatol. (2005) 14:719–26. 10.1111/j.1600-0625.2005.00363.x 16176279

[4] Zegarska B Pietkun K Zegarski W Bolibok P Wiśniewski M Roszek K. Air pollution, UV irradiation and skin carcinogenesis: what we know, where we stand and what is likely to happen in the future? Postep dermatologii i Alergol. (2017) 34:6–14. 10.5114/ada.2017.65616 28261026 PMC5329103

[5] Hahn ME Karchner SI Shapiro MA Perera SA. Molecular evolution of two vertebrate aryl hydrocarbon (dioxin) receptors (AHR1 and AHR2) and the PAS family. Proc Natl Acad Sci USA. (1997) 94:13743–8. 10.1073/pnas.94.25.13743 9391097 PMC28377

[6] Fujii-Kuriyama Y Mimura J. Molecular mechanisms of AhR functions in the regulation of cytochrome P450 genes. Biochem Biophys Res Commun. (2005) 338:311–7. 10.1016/j.bbrc.2005.08.162 16153594

[7] Safe S Cheng Y Jin UH. The aryl hydrocarbon receptor (AhR) as a drug target for cancer chemotherapy. Curr Opin Toxicol. (2017) 2:24–9. 10.1016/j.cotox.2017.01.012 28459113 PMC5407490

[8] Chahal HS Lin Y Ransohoff KJ Hinds DA Wu W Dai HJ. Genome-wide association study identifies novel susceptibility loci for cutaneous squamous cell carcinoma. Nat Commun. (2016) 7:12048. 10.1038/ncomms12048 27424798 PMC4960294

[9] Lv JW Zheng ZQ Wang ZX Zhou GQ Chen L Mao YP. Pan-cancer genomic analyses reveal prognostic and immunogenic features of the tumor melatonergic microenvironment across 14 solid cancer types. J Pineal Res. (2019) 66:1–13. 10.1111/jpi.12557 30638277

[10] Pollet M Shaik S Mescher M Frauenstein K Tigges J Braun SA. The AHR represses nucleotide excision repair and apoptosis and contributes to UV-induced skin carcinogenesis. Cell Death Differ. (2018) 25:1823–36. 10.1038/s41418-018-0160-1 30013037 PMC6180092

[11] Matsumoto Y Ide F Kishi R Akutagawa T Sakai S Nakamura M. Aryl hydrocarbon receptor plays a significant role in mediating airborne particulate-induced carcinogenesis in mice. Environ Sci Technol. (2007) 41:3775–80. 10.1021/es062793g 17547212

[12] Corre S Tardif N Mouchet N Leclair HM Boussemart L Gautron A. Sustained activation of the Aryl hydrocarbon Receptor transcription factor promotes resistance to BRAF-inhibitors in melanoma. Nat Commun. (2018) 9: 10.1038/s41467-018-06951-2 30429474 PMC6235830

[13] Liu Y Liang X Dong W Fang Y Lv J Zhang T. Tumor-repopulating cells induce PD-1 expression in CD8+ T cells by transferring kynurenine and AhR activation. Cancer Cell. (2018) 33:480–494.e7. 10.1016/j.ccell.2018.02.005 29533786

[14] Vitaliano PP Urbach F. The relative importance of risk factors in nonmelanoma carcinoma. Arch Dermatol. (1980) 116:454–6. 7369779

[15] Cadet J Sage E Douki T. Ultraviolet radiation-mediated damage to cellular DNA. Mutat Res. (2005) 571:3–17. 10.1016/j.mrfmmm.2004.09.012 15748634

[16] Roos WP Thomas AD Kaina B. DNA damage and the balance between survival and death in cancer biology. Nat Rev Cancer. (2016) 16:20–33. 10.1038/nrc.2015.2 26678314

[17] DiGiovanna JJ Kraemer KH. Shining a light on xeroderma pigmentosum. J Invest Dermatol. (2012) 132:785–96. 10.1038/jid.2011.426 22217736 PMC3279615

[18] Garibyan L Fisher DE. How sunlight causes melanoma. Curr Oncol Rep. (2010) 12:319–26. 10.1007/s11912-010-0119-y 20623386

[19] Fritsche E Schäfer C Calles C Bernsmann T Bernshausen T Wurm M. Lightening up the UV response by identification of the arylhydrocarbon receptor as a cytoplasmatic target for ultraviolet B radiation. Proc Natl Acad Sci USA. (2007) 104:8851–6. 10.1073/pnas.0701764104 17502624 PMC1885591

[20] Frauenstein K Sydlik U Tigges J Majora M Wiek C Hanenberg H. Evidence for a novel anti-apoptotic pathway in human keratinocytes involving the aryl hydrocarbon receptor, E2F1, and checkpoint kinase 1. Cell Death Differ. (2013) 20:1425–34. 10.1038/cdd.2013.102 23912710 PMC3770322

[21] Muthusamy V Piva TJ. The UV response of the skin: a review of the MAPK, NFkappaB and TNFalpha signal transduction pathways. Arch Dermatol Res. (2010) 302:5–17. 10.1007/s00403-009-0994-y 19756672

[22] Smith KJ Boyer JA Muku GE Murray IA Gowda K Desai D. Editor's highlight: Ah receptor activation potentiates neutrophil chemoattractant (C-X-C Motif) ligand 5 expression in keratinocytes and skin. Toxicol Sci. (2017) 160:83–94. 10.1093/toxsci/kfx160 28973351 PMC5837612

[23] Tanaka Y Uchi H Hashimoto-Hachiya A Furue M. Tryptophan photoproduct FICZ upregulates IL1A, IL1B, and IL6 expression via oxidative stress in keratinocytes. Oxid Med Cell Longev. (2018) 2018:9298052. 10.1155/2018/9298052 30595799 PMC6286782

[24] Buckman SY Gresham A Hale P Hruza G Anast J Masferrer J Pentland AP. COX-2 expression is induced by UVB exposure in human skin: implications for the development of skin cancer. Carcinogenesis. (1998) 19:723–9. 9635856 10.1093/carcin/19.5.723

[25] Loomis D Grosse Y Lauby-Secretan B Ghissassi F El Bouvard V Benbrahim-Tallaa L. The carcinogenicity of outdoor air pollution. Lancet Oncol. (2013) 14:1262–3. 10.1016/s1470-2045(13)70487-x 25035875

[26] Burnett RT1 Pope CA III Ezzati M Olives C Lim SS Mehta S. An integrated risk function for estimating the global burden of disease attributable to ambient fine particulate matter exposure. Environ Health Perspect. (2014) 122:397–403. 10.1289/ehp.1307049 24518036 PMC3984213

[27] Harrison RM Smith DJT Kibble AJ. What is responsible for the carcinogenicity of PM2.5? Occup Environ Med. (2004) 61:799–805. 10.1136/oem.2003.010504 15377764 PMC1740668

[28] Sanders CL Skinner C Gelman RA. Percutaneous absorption of 7, 10 14C-benzo[a]pyrene and 7, 12 14C-dimethylbenz[a]anthracene in mice. J Environ Pathol Toxicol Oncol. (1984) 7:25–34. 3098955

[29] Kao J Patterson FK Hall J Skin penetration and metabolism of topically applied chemicals in six mammalian species, including man: an *in vitro* study with benzo[a]pyrene and testosterone. Toxicol Appl Pharmacol. (1985) 81:502–16. 10.1016/0041-008X(85)90421-1 3936234

[30] Chu I Dick D Bronaugh R Tryphonas L. Skin reservoir formation and bioavailability of dermally administered chemicals in hairless guinea pigs. Food Chem Toxicol. (1996) 34:267–76. 10.1016/0278-6915(95)00112-3 8621108

[31] Rao MS Subbarao V Prasad JD Scarpelli DG. Carcinogenicity of 2,3,7,8-tetrachlorodibenzo-p-dioxin in the Syrian golden hamster. Carcinogenesis. (1988) 9:1677–9. 10.1093/carcin/9.9.1677 3409472

[32] Melendez-Colon VJ Luch A Seidel A Baird WM. Cancer initiation by polycyclic aromatic hydrocarbons results from formation of stable DNA adducts rather than apurinic sites. Carcinogenesis. (1999) 20:1885–91. 10506100 10.1093/carcin/20.10.1885

[33] Huang PY Balmain A. Modeling cutaneous squamous carcinoma development in the mouse. Cold Spring Harb Perspect Med. (2014) 4:a013623. 10.1101/cshperspect.a013623 25183851 PMC4143107

[34] Nassar D Latil M Boeckx B Lambrechts D Blanpain C. Genomic landscape of carcinogen-induced and genetically induced mouse skin squamous cell carcinoma. Nat Med. (2015) 21:946–54. 10.1038/nm.3878 26168291

[35] McCreery MQ Halliwill KD Chin D Delrosario R Hirst G Vuong P. Evolution of metastasis revealed by mutational landscapes of chemically induced skin cancers. Nat Med. (2015) 21:1514–20. 10.1038/nm.3979 26523969 PMC5094808

[36] Ginos MA Page GP Michalowicz BS Patel KJ Volker SE Pambuccian SE. Identification of a gene expression signature associated with recurrent disease in squamous cell carcinoma of the head and neck. Cancer Res. (2004) 64:55–63. 10.1158/0008-5472.CAN-03-2144 14729608

[37] Tormo D Ferrer A Gaffal E Wenzel J Basner-Tschakarjan E Steitz J. Rapid growth of invasive metastatic melanoma in carcinogen-treated hepatocyte growth factor/scatter factor-transgenic mice carrying an oncogenic CDK4 mutation. Am J Pathol. (2006) 169:665–72. 10.2353/ajpath.2006.060017 16877364 PMC1698803

[38] Shimizu Y Nakatsuru Y Ichinose M Takahashi Y Kume H Mimura J Fujii-Kuriyama Y Ishikawa T. Benzo[a]pyrene carcinogenicity is lost in mice lacking the aryl hydrocarbon receptor. Proc Natl Acad Sci USA. (2000) 97:779–82. 10.1073/pnas.97.2.779 10639156 PMC15407

[39] De Souza VRC Cabrera WK Galvan A Ribeiro OG De Franco M Vorraro F. Aryl hydrocarbon receptor polymorphism modulates DMBA-induced inflammation and carcinogenesis in phenotypically selected mice. Int J Cancer. (2009) 124:1478–82. 10.1002/ijc.24066 19065662

[40] Ide F Suka N Kitada M Sakashita H Kusama K Ishikawa T Skin and salivary gland carcinogenicity of 7,12-dimethylbenz[a]anthracene is equivalent in the presence or absence of aryl hydrocarbon receptor. Cancer Lett. (2004) 214:35–41. 10.1016/j.canlet.2004.04.014 15331171

[41] Guengerich FP. Cytochrome p450 and chemical toxicology. Chem Res Toxicol. (2008) 21:70–83. 10.1021/tx700079z 18052394

[42] Kleiner HE Vulimiri SV Reed MJ Uberecken A DiGiovanni J. Role of cytochrome P450 1a1 and 1b1 in the metabolic activation of 7,12-dimethylbenz[a]anthracene and the effects of naturally occurring furanocoumarins on skin tumor initiation. Chem Res Toxicol. (2002) 15:226–35. 10.1021/tx010151v 11849049

[43] Shimada T. Xenobiotic-metabolizing enzymes involved in activation and detoxification of carcinogenic polycyclic aromatic hydrocarbons. Drug Metab Pharmacokinet. (2006) 21:257–76. 10.2133/dmpk.21.257 16946553

[44] Xue P Fu J Zhou Y. The aryl hydrocarbon receptor and tumor immunity. Front Immunol. (2018) 9:286. 10.3389/fimmu.2018.00286 29487603 PMC5816799

[45] Feng S Cao Z Wang X. Role of aryl hydrocarbon receptor in cancer. Biochim Biophys Acta. (2013) 1836:197–210. 10.1016/j.bbcan.2013.05.001 23711559

[46] Lin JY Fisher DE. Melanocyte biology and skin pigmentation. Nature. (2007) 445:843–50. 10.1038/nature05660 17314970

[47] Jux B Kadow S Luecke S Rannug A Krutmann J Esser C. The aryl hydrocarbon receptor mediates UVB radiation-induced skin tanning. J Invest Dermatol. (2011) 131:203–10. 10.1038/jid.2010.269 20861855

[48] O'Donnell EF Kopparapu PR Koch DC Jang HS Phillips JL Tanguay RL. The Aryl hydrocarbon receptor mediates leflunomide-induced growth inhibition of melanoma cells. PLoS ONE. (2012) 7: 10.1371/journal.pone.0040926 22815870 PMC3398955

[49] Contador-Troca M Alvarez-Barrientos A Barrasa E Rico-Leo EM Catalina-Fernández I Menacho-Márquez M. The dioxin receptor has tumor suppressor activity in melanoma growth and metastasis. Carcinogenesis. (2013) 34:2683–93. 10.1093/carcin/bgt248 23843039

[50] Villano CM Murphy KA Akintobi A White LA. 2,3,7,8-tetrachlorodibenzo-p-dioxin (TCDD) induces matrix metalloproteinase (MMP) expression and invasion in A2058 melanoma cells. Toxicol Appl Pharmacol. (2006) 210:212–24. 10.1016/j.taap.2005.05.001 15982688

[51] Barretina J Caponigro G Stransky N Venkatesan K Margolin AA Kim S. The Cancer Cell Line Encyclopedia enables predictive modelling of anticancer drug sensitivity. Nature. (2012) 483:603–7. 10.1038/nature11003 22460905 PMC3320027

[52] Coit DG Thompson JA Algazi A Andtbacka R Bichakjian CK Carson WE. Melanoma, Version 2.2016, NCCN clinical practice guidelines in oncology. J Natl Compr Canc Netw. (2016) 14:450–73. 10.6004/jnccn.2016.0051 27059193

[53] Miller AJ Mihm MC. Melanoma. N Engl J Med. (2006) 355:51–65. 10.1056/NEJMra052166 16822996

[54] Luther C Swami U Zhang J Milhem M Zakharia Y. Advanced stage melanoma therapies: detailing the present and exploring the future. Crit Rev Oncol Hematol. (2019) 133:99–111. 10.1016/j.critrevonc.2018.11.002 30661664

[55] Colombino M Capone M Lissia A Cossu A Rubino C De Giorgi V. BRAF/NRAS mutation frequencies among primary tumors and metastases in patients with melanoma. J Clin Oncol. (2012) 30:2522–9. 10.1200/JCO.2011.41.2452 22614978

[56] Chapman PB Hauschild A Robert C Haanen JB Ascierto P Larkin J. Improved survival with vemurafenib in melanoma with BRAF V600E mutation. N Engl J Med. (2011) 364:2507–16. 10.1056/NEJMoa1103782 21639808 PMC3549296

[57] Hauschild A Grob JJ Demidov LV Jouary T Gutzmer R Millward M. Dabrafenib in BRAF-mutated metastatic melanoma: a multicentre, open-label, phase 3 randomised controlled trial. Lancet. (2012) 380:358–65. 10.1016/S0140-6736(12)60868-X 22735384

[58] Long GV Weber JS Infante JR Kim KB Daud A Gonzalez R. Overall survival and durable responses in patients with BRAF V600-mutant metastatic melanoma receiving dabrafenib combined with trametinib. J Clin Oncol. (2016) 34:871–8. 10.1200/JCO.2015.62.9345 26811525

[59] Müller J Krijgsman O Tsoi J Robert L Hugo W Song C. Low MITF/AXL ratio predicts early resistance to multiple targeted drugs in melanoma. Nat Commun. (2014) 5:5712. 10.1038/ncomms6712 25502142 PMC4428333

[60] Kong X Kuilman T Shahrabi A Boshuizen J Kemper K Song JY. Cancer drug addiction is relayed by an ERK2-dependent phenotype switch. Nature. (2017) 550:270–4. 10.1038/nature24037 28976960 PMC5640985

[61] Alexandrov LB Nik-Zainal S Wedge DC Aparicio SAJR Behjati S Biankin AV. Signatures of mutational processes in human cancer. Nature. (2013) 500:415–21. 10.1038/nature12477 23945592 PMC3776390

[62] Efremova M Finotello F Rieder D Trajanoski Z. Neoantigens generated by individual mutations and their role in cancer immunity and immunotherapy. Front Immunol. (2017) 8:1–8. 10.3389/fimmu.2017.01679 29234329 PMC5712389

[63] Rooney MS Shukla SA Wu CJ Getz G Hacohen N. Molecular and genetic properties of tumors associated with local immune cytolytic activity. Cell. (2015) 160:48–61. 10.1016/j.cell.2014.12.033 25594174 PMC4856474

[64] Rodić N Anders RA Eshleman JR Lin MT Xu H Kim JH PD-L1 expression in melanocytic lesions does not correlate with the BRAF V600E mutation. Cancer Immunol Res. (2015) 3:110–5. 10.1158/2326-6066.CIR-14-0145 25370533 PMC4324161

[65] Dong H Strome SE Salomao DR Tamura H Hirano F Flies DB. Tumor-associated B7-H1 promotes T-cell apoptosis: a potential mechanism of immune evasion. Nat Med. (2002) 8:793–800. 10.1038/nm730 12091876

[66] Hirano F Kaneko K Tamura H Dong H Wang S Ichikawa M. Blockade of B7-H1 and PD-1 by monoclonal antibodies potentiates cancer therapeutic immunity. Cancer Res. (2005) 65:1089–96. 15705911

[67] Robert C Long GV Brady B Dutriaux C Maio M Mortier L. Nivolumab in previously untreated melanoma without BRAF mutation. N Engl J Med. (2015) 372:320–30. 10.1056/NEJMoa1412082 25399552

[68] Liu Y Liang X Yin X Lv J Tang K Ma J. Blockade of IDO-kynurenine-AhR metabolic circuitry abrogates IFN-γ-induced immunologic dormancy of tumor-repopulating cells. Nat Commun. (2017) 8:15207. 10.1038/ncomms15207 28488695 PMC5436221

[69] Liu Y Lv J Liu J Liang X Jin X Xie J. STAT3/p53 pathway activation disrupts IFN-β-induced dormancy in tumor-repopulating cells. J Clin Invest. (2018) 128:1057–73. 10.1172/JCI96329 29431732 PMC5824876

[70] Wardhani LO Matsushita M Iwasaki T Kuwamoto S Nonaka D Nagata K Expression of the IDO1/TDO2-AhR pathway in tumor cells or the tumor microenvironment is associated with Merkel cell polyomavirus status and prognosis in Merkel cell carcinoma. Hum Pathol. (2019) 84:52–61. 10.1016/j.humpath.2018.09.003 30240768

[71] Sato Y Fujimura T Tanita K Lyu C Matsushita S Fujisawa Y. Malassezia-derived aryl hydrocarbon receptor ligands enhance the CCL20/ Th17/soluble CD163 pathogenic axis in extra-mammary Paget's disease. Exp Dermatol. (2019) 28:933–39. 10.1111/exd.13944 31001887

